# Association of living arrangements with all-cause mortality among older adults: a propensity score–matched cohort study

**DOI:** 10.1186/s12889-023-16749-7

**Published:** 2023-09-19

**Authors:** Lining Pu, Jingni Zhang, Xiaoxue He, Degong Pan, Huihui Wang, Xue Zhang, Xian Sun, Xiaojuan Liu, Shulan He, Jiangping Li

**Affiliations:** 1 Department of Epidemiology and Health Statistics, School of Public Health, Ningxia Medical University, Yinchuan, 750004 Ningxia Hui Autonomous Region China; 2 Department of Science and Education, Qinghai Provincial People’s Hospital, Xining, 810007 Qinghai Province China; 3 Key Laboratory of Environmental Factors and Chronic Disease Control, Ningxia Medical University, Yinchuan, 750004 Ningxia Hui Autonomous Region China

**Keywords:** All-cause mortality, Living arrangements, Older adults, Propensity score matching

## Abstract

**Background:**

Many studies exist on the living arrangements and health status of older adults, but the findings have been inconsistent. Therefore, we examined the relationship between living arrangements and all-cause mortality in older adults.

**Methods:**

This perspective study was based on the Chinese Longitudinal Healthy Longevity Survey (CLHLS) from 2011 to 2018. We used a sample aged 65 years and over included in the study in 2011. Propensity score matching was performed to minimize bias and Cox proportional hazards regression models were conducted.

**Results:**

A total of 7,963 participants were included. Of these, 1,383 were living alone, 6,424 were living with families, and 156 were living in nursing homes. In the propensity score-matched cohort, older adults living alone had a significantly lower risk of all-cause mortality than those living with families (hazard ratio 0.85; 95% confidence intervals 0.76 to 0.95). Living alone was prominently associated with a decline in mortality compared with living in nursing homes (hazard ratio 0.61; 95% confidence intervals 0.44 to 0.84). There was no significant difference in mortality between living in nursing homes and living with families (hazard ratio 1.19; 95% confidence intervals 0.89 to 1.60). Subgroup analyses indicated that there was no significant interaction with age, sex, education, or residence.

**Conclusions:**

The risk of all-cause mortality was significantly lower in older adults living alone than in those living with families or living in nursing homes. This article’s findings suggest the need to adopt multiple approaches to meet the needs of senior care services.

## Background

Living arrangements among older adults have attracted a great deal of attention with the acceleration of population aging in China. According to conventional perceptions, living with family members (who provide both physical and psychological support) remains the traditional living arrangement for older people [1, 2]. However, with economic development and social transformation, the economic independence and self-consciousness of older people have been enhanced and pension provision has increased, which has altered traditions so that preferences for living arrangements have changed [3, 4]. The proportion of rural Chinese aged 65 years and above living with family members fell from 71.3% in 1990 to 56.5% in 2010, while living alone rose rapidly from 27.1% to 42.2% [5]. In addition, with the improvement in the demand for pension services, the development of China’s nursing home industry has accelerated [6]. Therefore, whether living arrangements have an impact on the health status of older individuals is an important topic.

Studies on the relationship between living arrangements and mortality risk in older people are relatively abundant; however, these studies have limitations. These limitations have included generalizing the classification of living arrangements, not controlling for confounding factors, and the rare use of a variety of unusual methods to test the reliability of the results. Such limitations have led to doubts about the obtained results. A recent meta-analysis has shown that compared with not living alone, living alone increases mortality risk for individuals under 65 years of age, but not in people over 75 years of age [7]. Several studies have reported that living alone or living in nursing homes is associated with an increased risk of mortality [8–14], yet others have shown that people living alone have a lower risk of death than those living with others [15, 16]. Furthermore, several investigations have concluded that there is no association between living alone and the risk of mortality [17, 18]. From the existing evidence, we speculate that differences in findings between living arrangements and mortality may be influenced by factors such as age, sex, education, and health status. Therefore, it is necessary to control these covariables to further explore this relationship. In addition, although nursing homes are continuously evolving, the uneven quality of nursing home services has been controversial, and research on the mortality risk of older people in nursing homes is lacking. Thus, a need exists to investigate the relationship between living arrangements and mortality, with a view to providing better living arrangements for older adults.

Hence, we used propensity score matching to compare all-cause mortality in people aged 65 years and over living alone, living with families, and living in nursing homes. Our objective was to estimate the hazard ratios (HRs) for all-cause mortality for different living arrangements.

## Methods

### Data sources and study cohort

This cohort study used data from the Chinese Longitudinal Healthy Longevity Survey (CLHLS), which covered 22 of 31 provinces in China [19]. The CLHLS, which investigated the determinants of health and longevity of older adults in China, began in 1998 and has since been followed up in 2000, 2002, 2005, 2008, 2011, 2014, and 2018. During the CLHLS baseline survey, respondents were people of 80 years and over, and the age range after 2002 was adjusted to those over 65 years of age. The study design has been described in detail elsewhere [20, 21].

We used the sample aged 65 years and over included in the 2011 follow-up study to classify living arrangements as living alone, living with families, or living in a nursing home. All participants were followed up in 2014 and in 2018 to monitor vital status. Follow-up continued until death, loss of follow-up, or the end of the study. Participants without baseline characteristics or incomplete records were excluded.

### Variables

The primary exposures were living arrangements including living alone (living in a one-person household), living with families (living with a spouse or a cohabitee and possibly with others), and living in nursing homes (living in various types of endowment institutions). The potential confounding variables were age, sex, residence, education, smoking, drinking, exercise, self-rated health, pension, hypertension, heart disease, and diabetes, cognitive function, ADL, BMI, cancer, bronchitis, emphysema, asthma, and pneumonia.

### Study outcome

The study outcome was all-cause mortality. In the second and third surveys, information was collected on the participants’ survival status and date of death. Those still alive or lost to follow-up were censored at last follow-up.

### Statistical analysis

To minimize the effects of potential confounding factors, propensity score matching was performed. The propensity score was estimated using a logistic regression model with living arrangements as the dependent variable and covariates included as baseline characteristics. Using nearest neighbor-matching, a 1:1 matching was conducted on the propensity score with a maximum caliper of 0.02. Standardized mean differences were calculated in order to examine differences in baseline characteristics between groups before and after matching, with values less than 0.1 indicating an adequate balance between comparison groups [22].

We used Cox proportional hazards regression models to estimate the association between all-cause mortality and living alone, living with families, or living in nursing homes. The cumulative incidences were computed using the Kaplan–Meier method. HRs and 95% confidence intervals (CI) were estimated using Cox proportional hazards regression models.

Subgroup analyses were carried out by age (65–74 years, 75–84 years, and ≥ 85 years), sex (female and male), residence (urban and rural), education (some schooling and no schooling), smoking (yes and no), drinking (yes and no), exercise (yes and no), self-rated health (bad, fair, and good), pension (yes and no), hypertension (yes and no), heart disease (yes and no), and diabetes (yes and no). Based on a likelihood ratio test, interaction analysis was used to test for differences between subgroups. Several sensitivity analyses were conducted to evaluate the robustness of the findings. First, including baseline characteristics in the unmatched cohort to estimate the HRs of study outcomes. Second, cognitive function, ADL, BMI, cancer, bronchitis, emphysema, asthma, and pneumonia were added to the variables mentioned in the first point to estimate the HRs of study outcomes after matching. Third, we excluded individuals who died within 3 months from the start of the study.

All statistical analyses were performed using StataMP 17 version and R4.2.1 statistical software. A two-tailed *P* < 0.05 was used to determine statistical significance.

## Results

### Study population and baseline characteristics

Of the 9,765 participants between 2011 and 2018, a total of 7,963 participants were identified by excluding 1,802 participants who had incomplete data records. A total of 1,383 were living alone, 6,424 were living with families, and 156 were living in nursing homes. After propensity score matching, living alone vs. living with families included 1,381 participants in each group, living alone vs. living in nursing homes included 141 participants in each group, and living in nursing homes vs. living with families included 152 participants in each group.

Tables 1, 2 and 3 show the baseline characteristics of the groups before and after the propensity score matching. There were no significant differences in most baseline features between the two groups after propensity score matching, as each standardized mean difference value was less than 0.1. In the matched cohort of living alone vs. living with families, the mean age of participants was 84.9 ± 9.9 years for living alone and 84.9 ± 10.5 years for living with families (36.0% and 36.6% were literate, respectively). Self-rated excellent health was 42.9% and 44.5%, respectively. As for the chronic diseases, 30.3% and 28.3% of participants had hypertension, 3.3% and 3.7% had diabetes, and 11.7% and 10.0% had heart diseases, respectively.

**Table 1:** Baseline characteristics of older adults living alone vs. living with families before and after propensity-score matching

	Before matching			After matching		
	Living alone	Living with families	Standardized Mean Difference	Living alone	Living with families	Standardized Mean Difference
No. of participants	1383	6424		1381	1381	
Age, mean (SD), y	84.9 ± 9.9	84.9 ± 11.2	0.00	84.93 ± 9.9	84.91 ± 10.5	0.00
Sex						
Male	549(39.7%)	3069(47.8%)	0.16	549(39.8%)	540(39.1%)	0.01
Female	834(60.3%)	3355(52.2%)	0.16	832(60.2%)	841(60.9%)	0.01
Residence						
Urban	584(42.2%)	3125(48.6%)	0.13	584(42.3%)	577(41.8%)	0.01
Rural	799(57.8%)	3299(51.4%)	0.13	797(57.7%)	804(58.2%)	0.01
Education						
Some schooling	497(35.9%)	2928(45.6%)	0.20	497(36.0%)	506(36.6%)	0.01
No schooling	886(64.1%)	3496(54.4%)	0.20	884(64.0%)	875(63.4%)	0.01
Smoking						
Yes	221(16.0%)	1245(19.4%)	0.09	221(16.0%)	213(15.4%)	0.02
No	1162(84.0%)	5179(80.6%)	0.09	1160(84.0%)	1168(84.6%)	0.02
Drinking						
Yes	220(15.9%)	1158(18.0%)	0.06	220(15.9%)	189(13.7%)	0.06
No	1163(84.1%)	5266 (82.0)	0.06	1161(84.1%)	1192(86.3%)	0.06
Exercise						
Yes	466(33.7%)	2319(36.1%)	0.05	464(33.6%)	463(33.5%)	0.00
No	917(66.3%)	4105 (63.9)	0.05	917(66.4%)	918(66.5%)	0.00
Self-rated health						
Bad	248(17.9%)	1080(16.8%)	0.03	246 (17.8%)	230(16.7%)	0.03
Fair	542(39.2%)	2393(37.3%)	0.04	542 (39.2%)	537 (38.9%)	0.01
Good	593(42.9%)	2951(45.9%)	0.06	593(42.9%)	614(44.5%)	0.03
Hypertension						
Yes	421(30.4%)	1863(29.0%)	0.03	419(30.3%)	391(28.3%)	0.04
No	962(69.6%)	4561 (71.0)	0.03	962(69.7%)	990 (71.7%)	0.04
Diabetes						
Yes	46(3.3%)	298(4.6%)	0.07	46(3.3%)	51 ( 3.7%)	0.02
No	1337(96.7)	6126 (95.4)	0.07	1335(96.7%)	1330(96.3%)	0.02
Heart diseases						
Yes	162(11.7%)	836(13.0%)	0.04	162(11.7%)	138(10.0%)	0.05
No	1221(88.3%)	5588(87.0%)	0.04	1219(88.3%)	1243(90.0%)	0.05
Pension						
Yes	192(13.9%)	1381(21.5%)	0.20	192(13.9%)	187 (13.5%)	0.01
No	1191(86.1%)	5043(78.5%)	0.20	1189(86.1%)	1194(86.5%)	0.01

**Table 2:** Baseline characteristics of older adults living alone vs. living in nursing homes before and after propensity-score matching

	Before matching			After matching		
	Living alone	Living in nursing homes	Standardized Mean Difference	Living alone	Living in nursing homes	Standardized Mean Difference
No. of participants	1383	156		141	141	
Age, mean (SD), y	84.9 ± 9.9	90.9 ± 9.9	0.61	89.05 ± 9.6	89.79 ± 9.6	0.07
Sex						
Male	549(39.7%)	71(45.5%)	0.12	72(51.1%)	66(46.8%)	0.09
Female	834(60.3%)	85(54.5%)	0.12	69(48.9%)	75(53.2%)	0.09
Residence						
Urban	584(42.2%)	113(72.4%)	0.64	99(70.2%)	98(69.5%)	0.02
Rural	799(57.8%)	43(27.6%)	0.64	42(29.8%)	43(30.5%)	0.02
Education						
Some schooling	497(35.9%)	63(40.4%)	0.09	52(36.9%)	57(40.4%)	0.07
No schooling	886(64.1%)	93(59.6%)	0.09	89(63.1%)	84(59.6%)	0.07
Smoking						
Yes	221(16.0%)	23(14.7%)	0.04	22(15.6%)	21(14.9%)	0.02
No	1162 (84.0%)	133(85.3%)	0.04	119(84.4%)	120(85.1%)	0.02
Drinking						
Yes	220(15.9%)	17(10.9%)	0.15	13(9.2%)	17(12.1%)	0.09
No	1163(84.1%)	139(89.1%)	0.15	128(90.8%)	124(87.9%)	0.09
Exercise						
Yes	466(33.7%)	63(40.4%)	0.14	54(38.3%)	57(40.4%)	0.04
No	917(66.3%)	93(59.6%)	0.14	87(61.7%)	84(59.6%)	0.04
Self-rated health						
Bad	248(17.9%)	35(22.4%)	0.07	33(23.4%)	30(21.3%)	0.05
Fair	542(39.2%)	54(34.6%)	0.16	51(36.2%)	48(34.0%)	0.05
Good	593(42.9%)	67(42.9%)	0.11	57(40.4%)	63(44.7%)	0.09
Hypertension						
Yes	421(30.4%)	58(37.2%)	0.14	53(37.6%)	51(36.2%)	0.03
No	962(69.6%)	98(62.8%)	0.14	88(62.4%)	90(63.8%)	0.03
Diabetes						
Yes	46(3.3)	4(2.6)	0.05	3(2.1%)	4(2.8%)	0.05
No	1337(96.7)	152(97.4)	0.05	138(97.9%)	137(97.2%)	0.05
Heart diseases						
Yes	162(11.7%)	25(16.0%)	0.12	21(14.9%)	22(15.6%)	0.02
No	1221(88.3%)	131(84.0%)	0.12	120(85.1%)	119(84.4%)	0.02
Pension						
Yes	192(13.9%)	51(32.7%)	0.46	41(29.1%)	43(30.5%)	0.03
No	1191(86.1)	105(67.3)	0.46	100(70.9%)	98(69.5%)	0.03

**Table 3:** Baseline characteristics of older adults living in nursing homes vs. living with families before and after propensity-score matching

	Before matching			After matching		
	Living in nursing homes	Living with families	Standardized Mean Difference	Living in nursing homes	Living with families	Standardized Mean Difference
No. of participants	156	6424		152	152	
Age, mean (SD), y	90.9 ± 9.9	84.9 ± 11.2	0.56	90.6 ± 9.8	90.5 ± 10.7	0.01
Sex						
Male	71(45.5%)	3069(47.8%)	0.03	70(46.1%)	75(49.3%)	0.07
Female	85(54.5%)	3355(52.2%)	0.03	82(53.9%)	77(50.7%)	0.07
Residence						
Urban	113(72.4%)	3125(48.6%)	0.50	109(71.7%)	108(71.1%)	0.02
Rural	43(27.6%)	3299(51.4%)	0.50	43(28.3%)	44(28.9%)	0.02
Education						
Some schooling	63(40.4%)	2928(45.6%)	0.11	61(40.1%)	66(43.4%)	0.07
No schooling	93(59.6%)	3496(54.4%)	0.11	91(59.9%)	86(56.6%)	0.07
Smoking						
Yes	23(14.7%)	1245(19.4%)	0.12	23(15.1%)	20(13.2%)	0.06
No	133(85.3%)	5179 (80.6%)	0.12	129(84.9%)	132(86.8%)	0.06
Drinking						
Yes	17(10.9%)	1158(18.0%)	0.20	17(11.2%)	13(8.6%)	0.09
No	139(89.1%)	5266 (82.0)	0.20	135(88.8%)	139(91.4%)	0.09
Exercise						
Yes	63(40.4%)	2319(36.1%)	0.09	60(39.5%)	56(36.8%)	0.05
No	93(59.6%)	4105 (63.9)	0.09	92(60.5%)	96(63.2%)	0.05
Self-rated health						
Bad	35(22.4%)	1080(16.8%)	0.14	33(21.7%)	27(17.8%)	0.10
Fair	54(34.6%)	2393(37.3%)	0.06	53(34.9%)	58(38.2%)	0.07
Good	67(42.9%)	2951(45.9%)	0.06	66(43.3%)	67(44.1%)	0.02
Hypertension						
Yes	58(37.2%)	1863(29.0%)	0.17	55(36.2%)	65(42.8%)	0.14
No	98(62.8%)	4561 (71.0)	0.17	97(63.8%)	87(57.2%)	0.14
Diabetes						
Yes	4(2.6)	298(4.6%)	0.11	4(2.6%)	4(2.6%)	0.00
No	152(97.4)	6126 (95.4)	0.11	148(97.4%)	148(97.4%)	0.00
Heart diseases						
Yes	25(16.0%)	836(13.0%)	0.09	24(15.8%)	23(15.1%)	0.02
No	131(84.0%)	5588(87.0%)	0.09	128(84.2%)	129(84.9%)	0.02
Pension						
Yes	51(32.7%)	1381(21.5%)	0.25	49(32.2%)	43(28.3%)	0.09
No	105(67.3)	5043 (78.5%)	0.25	103(67.8%)	109(71.7%)	0.09

In the matched cohort of living alone vs. living in nursing homes, the mean age of participants was 89.1 ± 9.6 years for living alone and 89.9 ± 9.6 years for living in nursing homes (36.9% and 40.4% were literate, respectively). Self-reported excellent health was 40.4% and 44.7%, respectively. As for chronic diseases, 37.6% and 36.2% of participants had hypertension, 2.1% and 2.8% had diabetes, and 14.9% and 15.6% had heart diseases, respectively. Detailed baseline characteristics of the participants living in nursing homes vs. living with families are listed in Table 3.

### Association of living arrangements and mortality

**Fig. 1:**
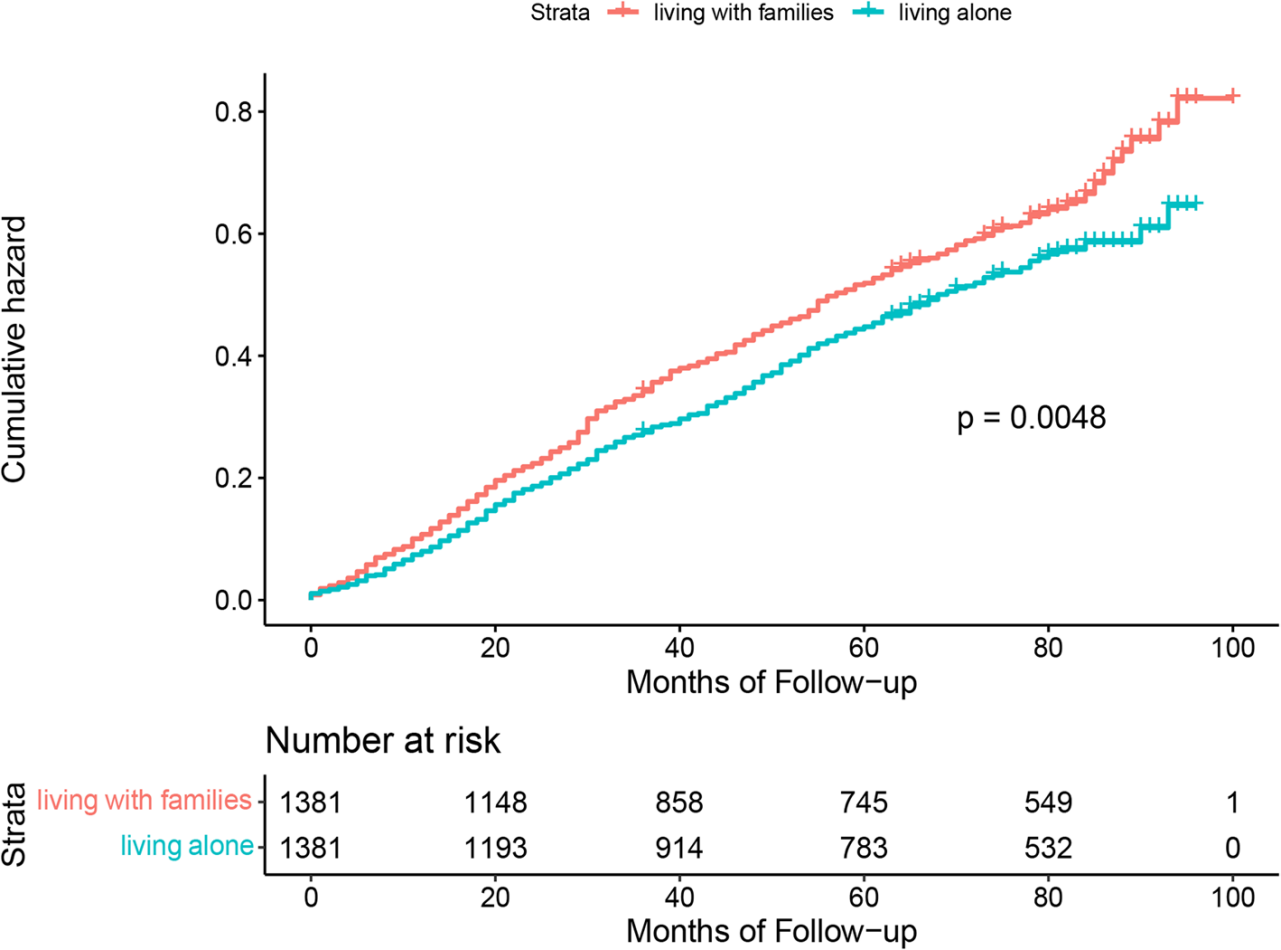
Kaplan–meier cumulative incidence plots for living alone vs. living with families in the matched cohort

**Fig. 2:**
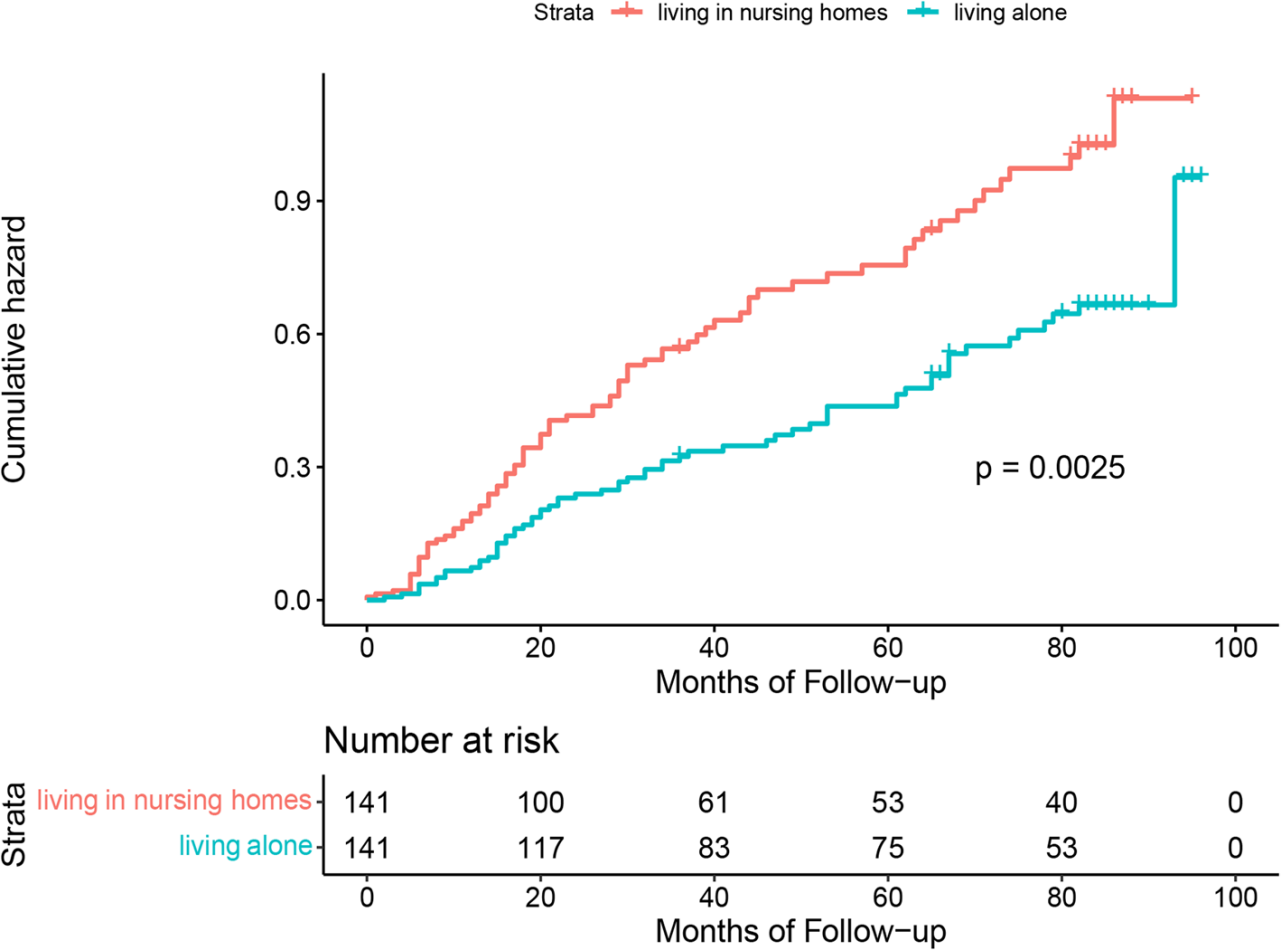
Kaplan–meier cumulative incidence plots for living alone vs. living in nursing homes in the matched cohort

**Fig. 3:**
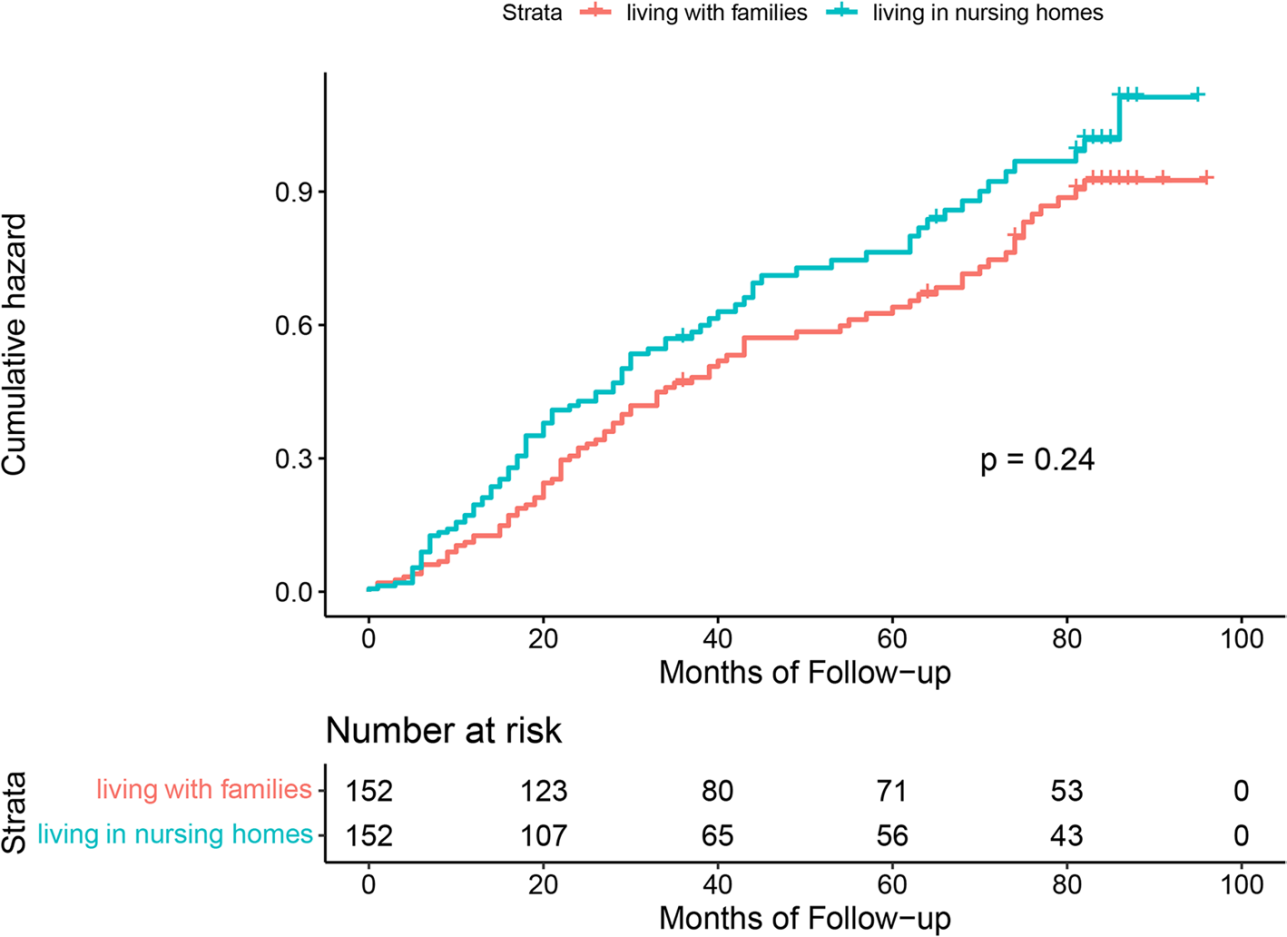
Kaplan–meier cumulative incidence plots for living in nursing homes vs. living with families in the matched cohort

**Fig. 4:**
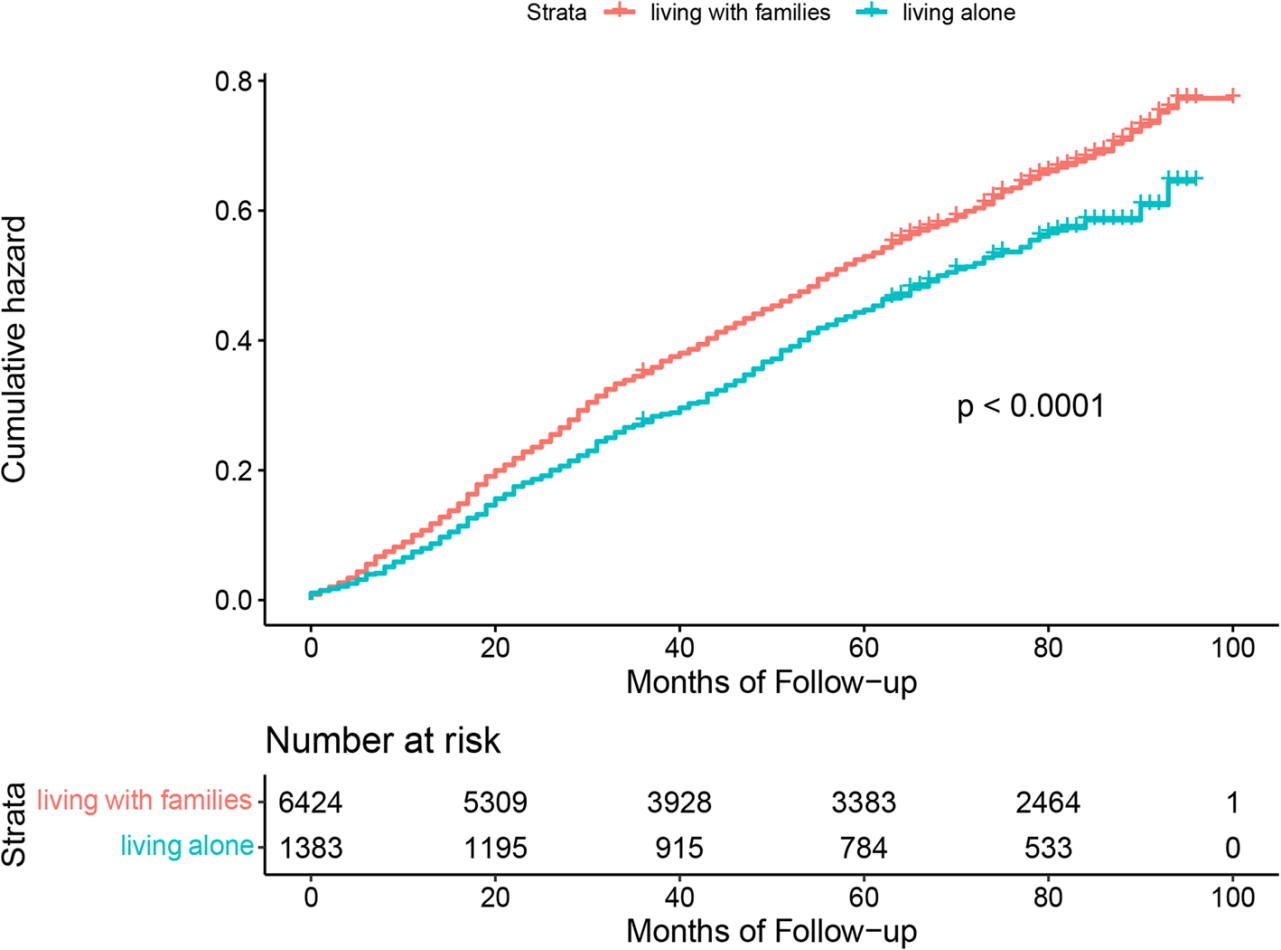
Kaplan–meier cumulative incidence plots for living alone vs. living with families in the unmatched cohort

**Fig. 5:**
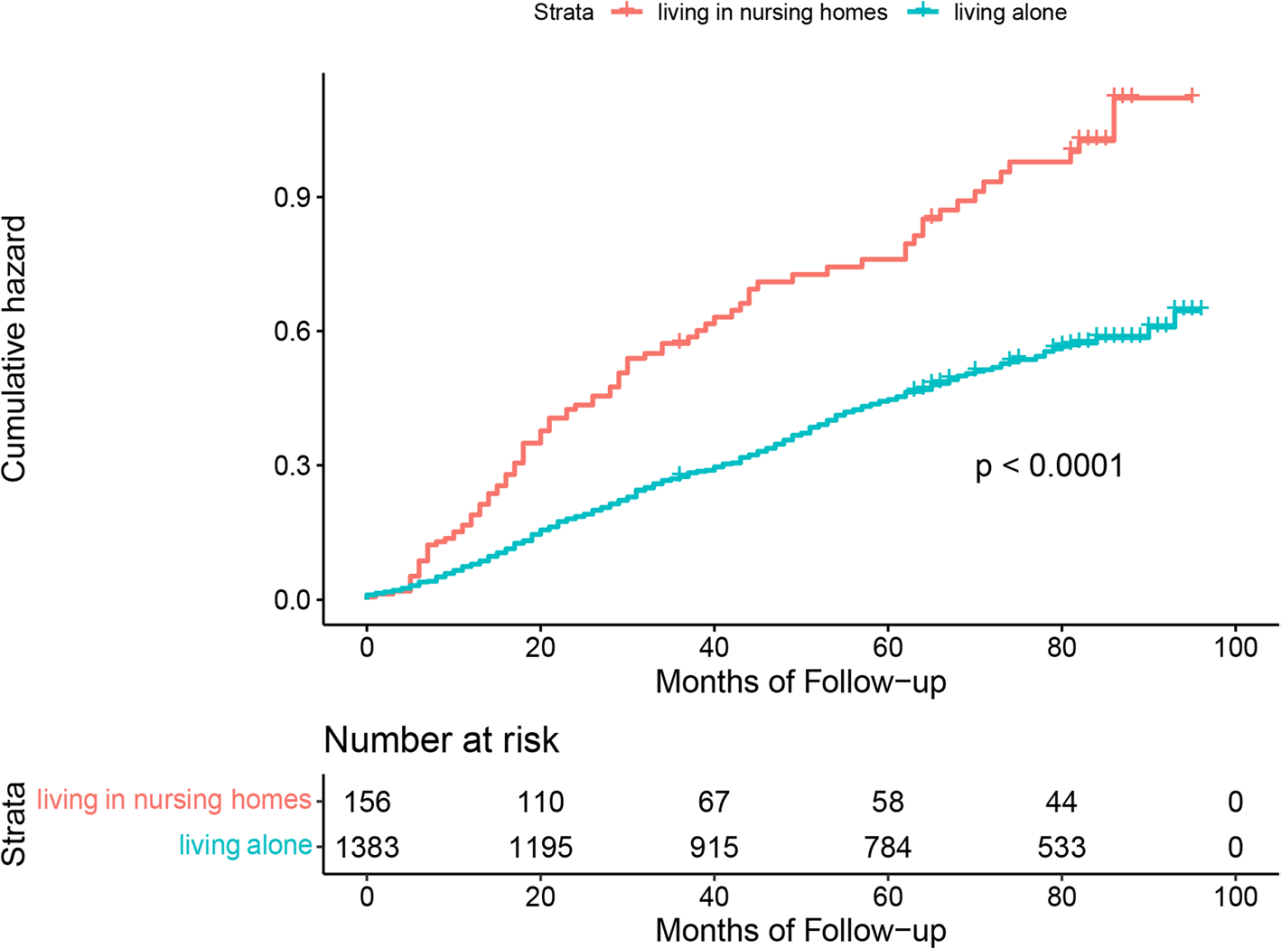
Kaplan–meier cumulative incidence plots for living alone vs. living in nursing homes in the unmatched cohort

**Fig. 6:**
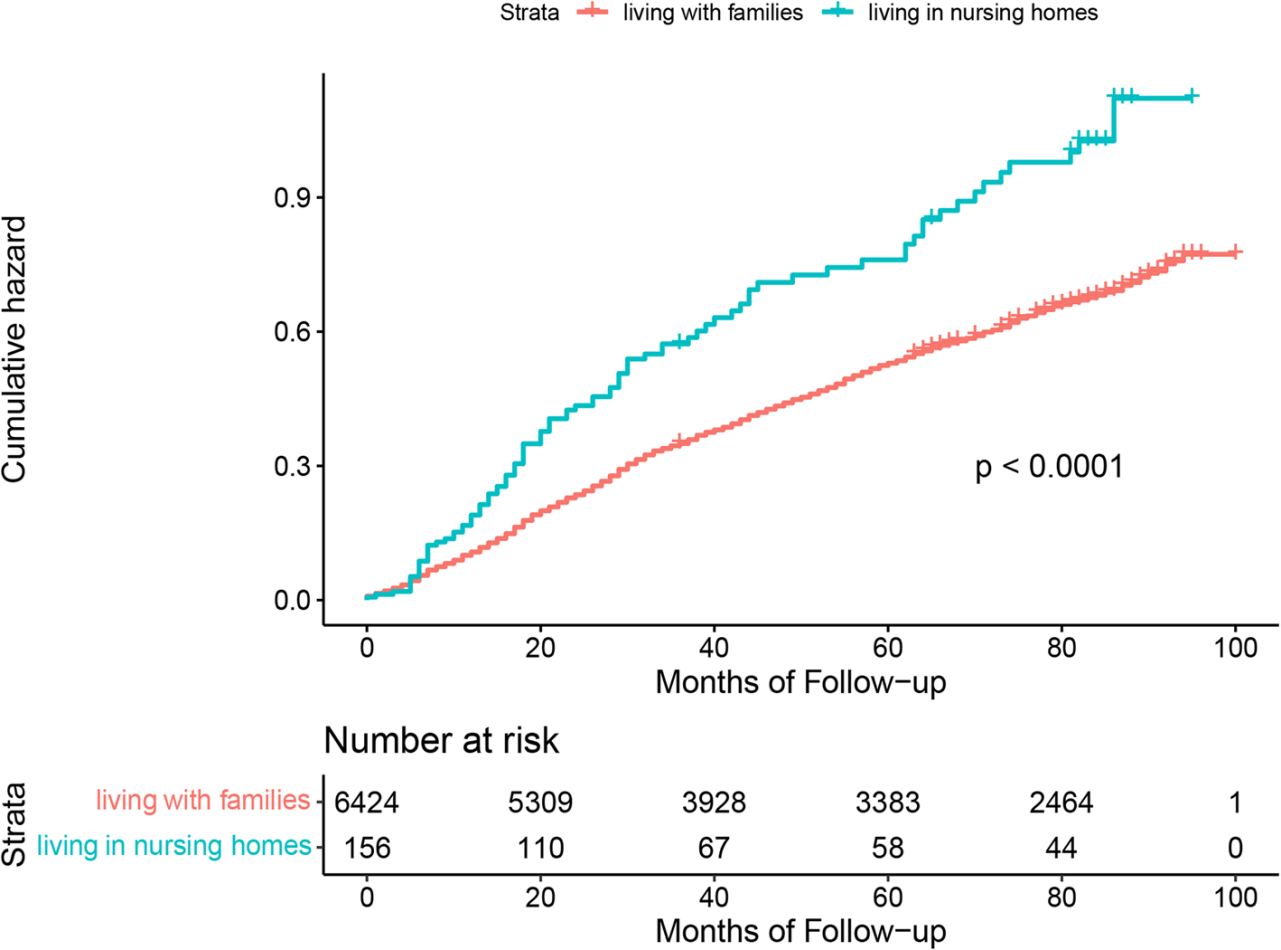
Kaplan–meier cumulative incidence plots for living in nursing homes vs. living with families in the unmatched cohort

Figures 1, 2 and 3 show the results of Kaplan–Meier analysis between living arrangements and mortality following propensity score matched analysis. Compared with living with families, older adults living alone had a significantly lower risk of all-cause mortality (HR 0.85; 95% CI 0.76 to 0.95). Similarly, living alone was prominently associated with a decline in mortality in the living alone vs. living in nursing homes cohort (HR 0.61; 95% CI 0.44 to 0.84). There was an insignificant difference in all-cause mortality for older adults living in nursing homes or living with families (HR 1.19; 95% CI 0.89 to 1.60). Kaplan–Meier plots are presented in Figs. 4, 5 and 6 for the unmatched cohorts.

### Subgroup and sensitivity analyses

**Fig. 7:**
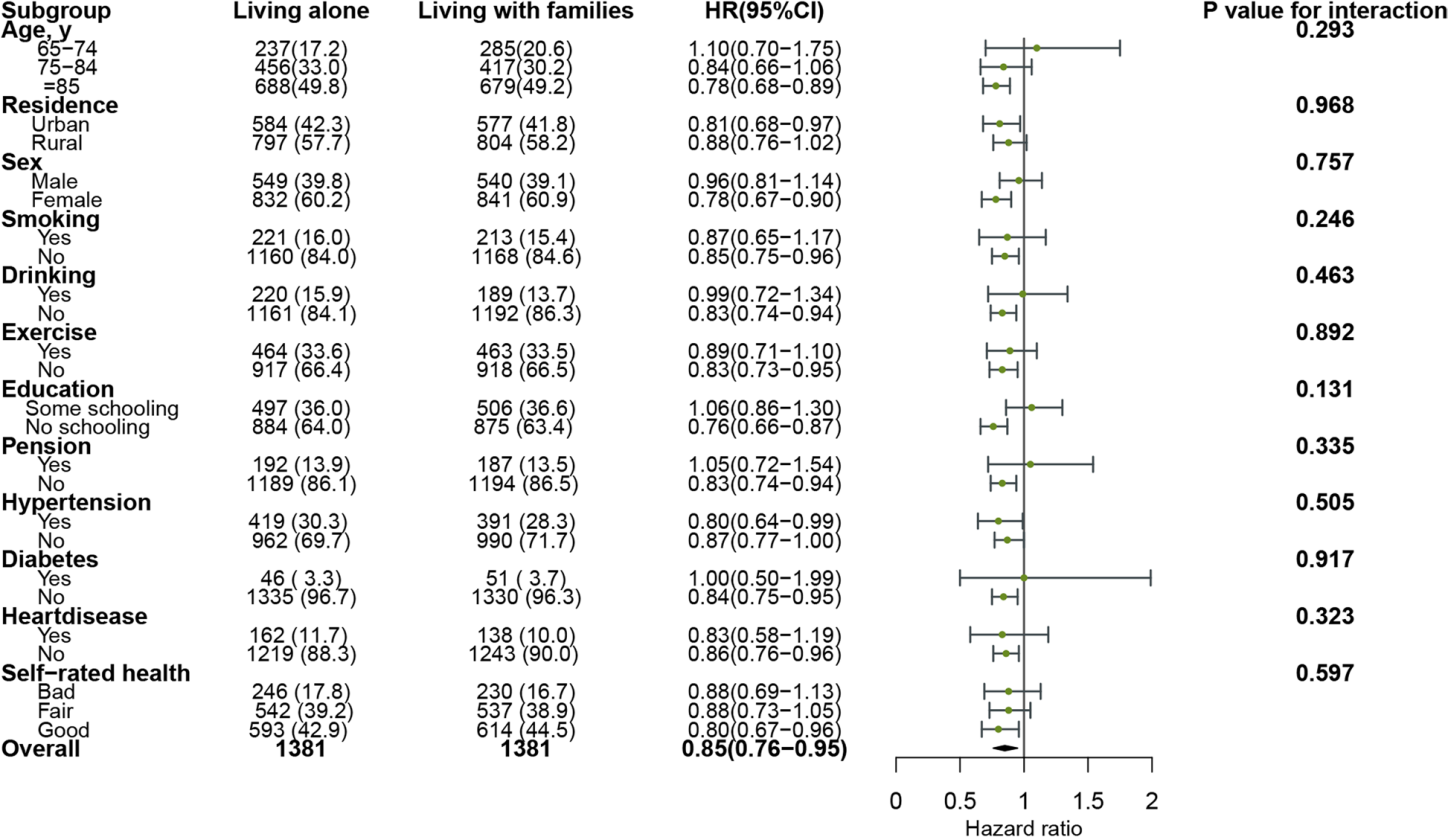
Subgroup-specific associations of living alone vs. living with families with all-cause mortality

**Fig. 8:**
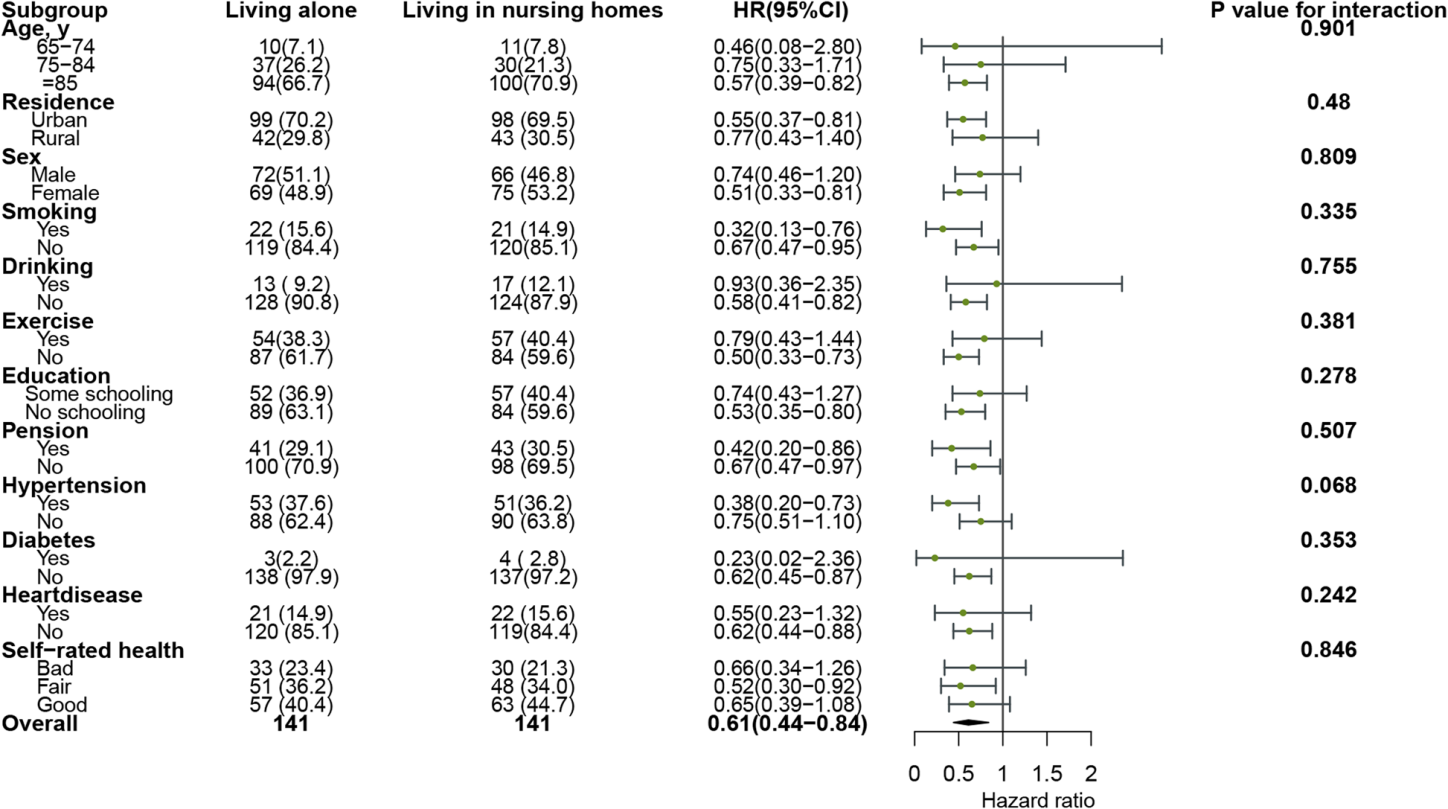
Subgroup-specific associations of living alone vs. living in nursing homes with all-cause mortality

There was no evidence for an interaction between living alone vs. living with families and all-cause mortality for age (*P*-value for interaction 0.293), sex (*P*-value for interaction 0.757), and education (*P*-value for interaction 0.131) (see Fig. 7). Residence (*P*-value for interaction 0.480), smoking (*P*-value for interaction 0.335), and exercise (*P*-value for interaction 0.381) did not achieve statistical significance between living alone vs. living in nursing homes and all-cause mortality (see Fig. 8). The sensitivity analyses consistently indicated that participants living alone had a significantly reduced the risk of all-cause mortality compared to living in nursing homes (see Table 4).

**Table 4:** Sensitivity analyses

Group	Approaches^a^	Hazard ratio (95% CI)
Living alone VS Living with families	unmatched cohort	0.84(0.76–0.91)
	matched cohort	0.99(0.85–1.15)
	3-month died	0.86(0.76–0.96)
Living alone VS Living in nursing homes	unmatched cohort	0.52(0.42–0.65)
	matched cohort	0.55(0.34–0.88)
	3-month died	0.62(0.44–0.86)

^a^unmatched cohort: covariates including age, sex, residence, education, smoking, drinking, exercise, self-rated health, pension, hypertension, heart disease, and diabetes; matched cohort: covariates including age, sex, residence, education, smoking, drinking, exercise, self-rated health, pension, hypertension, heart disease, diabetes, cognitive function, ADL, BMI, cancer, bronchitis, emphysema, asthma, and pneumonia

## Discussion

In this study, we revealed that older people who live alone have a lower risk of all-cause mortality than those living with families or living in nursing homes. In addition, we found no statistical difference in all-cause mortality between living in nursing homes and living with families.

The finding that older people living alone have a lower risk of mortality is in agreement with several previous studies [23, 24], but in opposition to the findings of other reports [25–30]. One suggestion is that people living alone are more likely to have lost a partner through divorce or death, and this exposes older adults to stress and loss of support, which puts them at greater risk of death [27]. There is no denying that in the traditional Chinese patrilineal culture with importance placed on filial piety, the disadvantages of living alone could be magnified. However, the following should be noted. Firstly, with rural revitalization and government subsidies, older adults living alone can be more independent and have better coping mechanisms for survival than those whose main source of income is their families. Older people living alone are able to control their finances independently, without interference from family members, which may have a positive psychological effect. Secondly, the preferences and opinions of young people are very different from those of their parents, and intergenerational conflicts living together are common, which can cause emotional stress for older adults. Thirdly, based on the current economic pressure and family structure in China, we suspect that there may be situations in which older adults need to take care of their grandchildren during the working week, which can be a burden.

One study concluded that living in nursing homes contributed to an increased risk of death when compared with living with families [31]. Here and in previous studies [28, 32], no significant differences were found between these two groups after controlling for potential confounders. This finding may be related to the personality of the individual older person. It has been reported that if older individuals’ living arrangements are consistent with their preferred living arrangements, the outcome is positive. For example, for older adults who like to be socially active and enjoy the recreational activities in nursing homes, living in a nursing home is associated with the likelihood of increased life satisfaction [33]. We, therefore, speculate that for older adults who are attached to their families and enjoy family life, living with families is beneficial. In addition, in both our study and a longitudinal study by Feng et al., living alone was associated with a lower risk of death compared with living in an institution [34]. In view of these results, we have the following thoughts. The findings may reflect the infancy of China’s pension institutions industry, as the senior care service system is not robust, and the quality of nursing home services varies. In the future, with the gradual increase of empty nest families and China’s aging population, nursing homes are expected to become a better choice for older people, as the quality and standards related to this innovative pension care service model grow.

It is worth mentioning that both our study and that of Ng et al. found that older people living alone had no more chronic disease incidence or physical function disability than their peers [35]. Wang et al. showed that older adults living with families were more vulnerable to becoming disabled than those living alone [36]. We speculate that living with family members may increase dependency compared with living alone, which could accelerate age-related loss of physical ability and thus increases the risk of illness and death, which is consistent with the conclusions of Li et al. [30]. By contrast, people living alone are more likely to be self-reliant and undertake some necessary physical exercise. Additionally, living alone is significantly positively correlated with mortality in men, but not in women [25, 37]. However, the present study showed that the relationship between living arrangements and mortality risk did not interact with sex. This discrepancy between studies could result from different types of study design or confounding factors. No consensus has yet been reached.

There were several limitations to this study. First, living arrangements were obtained from baseline; therefore, we did not take into account changes in living arrangements over time. Second, we were unable to obtain information on the specific causes of death in older adults and only explored all-cause mortality. Third, although we matched a number of variables using propensity score matching, variables not included might have affected the relationship between living arrangements and mortality risk.

The major advantage of our study was to divide the living arrangements into three categories for pairwise comparison. Compared with the general classification of living alone or not, our design was closer to the actual living arrangements of Chinese older adults. Thus, we could explore the similarities and differences between various living arrangements. Another strength was the use of propensity score matching to control for potential confounders. This study suggests that the perception of older adults who live alone as being a vulnerable risk group could be incorrect. We should fully respect the preferences of older people regarding their living arrangements. Education is necessary to eliminate the fear of older individuals that their choice of living arrangements may directly affect their risk of death. It is important for society as a whole and its health care system to promote awareness of the real needs of the new era of aging and health in old age, and to strive to create a positive atmosphere in which multiple approaches to aging can be adopted.

## Conclusion

The current study used propensity score matching to reveal that older adults living alone may have a lower risk of all-cause mortality than those living with families or in nursing homes. There was an insignificant difference in all-cause mortality between older adults living in nursing homes and those living with families. The study findings provide a new explanation for the relationship between living arrangements and all-cause mortality, which can inform the development of the pension service system.

## Data Availability

Publicly available datasets were analyzed in this study. This data can be found at: https://opendata.pku.edu.cn.

## Abbreviations

CLHLS: Chinese longitudinal healthy longevity survey
HRs: Hazard ratios
CI: Confidence intervals
